# Plasma Free Amino Acid Profiling of Five Types of Cancer Patients and Its Application for Early Detection

**DOI:** 10.1371/journal.pone.0024143

**Published:** 2011-09-07

**Authors:** Yohei Miyagi, Masahiko Higashiyama, Akira Gochi, Makoto Akaike, Takashi Ishikawa, Takeshi Miura, Nobuhiro Saruki, Etsuro Bando, Hideki Kimura, Fumio Imamura, Masatoshi Moriyama, Ichiro Ikeda, Akihiko Chiba, Fumihiro Oshita, Akira Imaizumi, Hiroshi Yamamoto, Hiroshi Miyano, Katsuhisa Horimoto, Osamu Tochikubo, Toru Mitsushima, Minoru Yamakado, Naoyuki Okamoto

**Affiliations:** 1 Molecular Pathology and Genetics Division, Kanagawa Cancer Center, Yokohama, Japan; 2 Department of Thoracic Surgery, Osaka Medical Center for Cancer and Cardiovascular Diseases, Osaka, Japan; 3 Department of Gastroenterological Surgery, Transplant and Surgical Oncology, Okayama University Graduate School of Medicine, Dentistry, and Pharmaceutical Sciences, Okayama, Japan; 4 Department of Gastrointestinal Surgery, Kanagawa Cancer Center, Yokohama, Japan; 5 Department of Breast and Thyroid Surgery, Yokohama City University Medical Center, Yokohama, Japan; 6 Department of Urology, Kanagawa Cancer Center, Yokohama, Japan; 7 Department of Anesthesia, Gunma Prefectural Cancer Center, Ohta, Japan; 8 Division of Gastric Surgery, Shizuoka Prefectural Cancer Center, Nagaizumi, Japan; 9 Division of Thoracic Diseases, Chiba Prefectural Cancer Center, Chiba, Japan; 10 Department of Pulmonary Oncology, Osaka Medical Center for Cancer and Cardiovascular Diseases, Osaka, Japan; 11 Department of Urology, Yokohama Municipal Citizen's Hospital, Yokohama, Japan; 12 Department of Urology, Yokohama Minami Kyosai Hospital, Yokohama, Japan; 13 Department of Breast and Thyroid Surgery, Kanagawa Cancer Center, Yokohama, Japan; 14 Department of Thoracic Oncology, Kanagawa Cancer Center, Yokohama, Japan; 15 Institute for Innovation, Ajinomoto, Co., Inc., Kawasaki, Japan; 16 Computational Biology Research Center, National Institute of Advanced Industrial Science and Technology, Tokyo, Japan; 17 Kanagawa Health Service Association, Yokohama, Japan; 18 Department of Gastroenterology, Kameda Medical Center Makuhari, Chiba, Japan; 19 Center for Multiphasic Health Testing and Services, Mitsui Memorial Hospital, Tokyo, Japan; 20 Department of Epidemiology, Kanagawa Cancer Center, Yokohama, Japan; Sun Yat-sen University Cancer Center, China

## Abstract

**Background:**

Recently, rapid advances have been made in metabolomics-based, easy-to-use early cancer detection methods using blood samples. Among metabolites, profiling of plasma free amino acids (PFAAs) is a promising approach because PFAAs link all organ systems and have important roles in metabolism. Furthermore, PFAA profiles are known to be influenced by specific diseases, including cancers. Therefore, the purpose of the present study was to determine the characteristics of the PFAA profiles in cancer patients and the possibility of using this information for early detection.

**Methods and Findings:**

Plasma samples were collected from approximately 200 patients from multiple institutes, each diagnosed with one of the following five types of cancer: lung, gastric, colorectal, breast, or prostate cancer. Patients were compared to gender- and age- matched controls also used in this study. The PFAA levels were measured using high-performance liquid chromatography (HPLC)–electrospray ionization (ESI)–mass spectrometry (MS). Univariate analysis revealed significant differences in the PFAA profiles between the controls and the patients with any of the five types of cancer listed above, even those with asymptomatic early-stage disease. Furthermore, multivariate analysis clearly discriminated the cancer patients from the controls in terms of the area under the receiver-operator characteristics curve (AUC of ROC >0.75 for each cancer), regardless of cancer stage. Because this study was designed as case-control study, further investigations, including model construction and validation using cohorts with larger sample sizes, are necessary to determine the usefulness of PFAA profiling.

**Conclusions:**

These findings suggest that PFAA profiling has great potential for improving cancer screening and diagnosis and understanding disease pathogenesis. PFAA profiles can also be used to determine various disease diagnoses from a single blood sample, which involves a relatively simple plasma assay and imposes a lower physical burden on subjects when compared to existing screening methods.

## Introduction

Several minimally-invasive, easy-to-use cancer diagnostic methods using peripheral blood or urine samples have recently been developed to ease the physical burden on patients and to reduce the costs and time involved [1], [2], [3], [4], [5], [6], [7], [8]. Rapid advances have been made in cancer diagnosis and prognosis methods based on metabolome analysis [3], [9], [10], [11], [12], [13], [14], which frequently involves the use of multivariate analysis techniques, such as computer-aided, machine-learning systems for data mining.

Although metabolome analysis is a promising approach in screening for diseases such as cancer, some practical limitations remain. These include the necessity to measure a huge number of metabolites [15], [16], [17], data-redundancy problems, including the false-discovery rate (FDR) and overfitting, and cost constraints. One approach to overcoming these problems is “focused metabolomics”, which limits the objects of the analysis to those that play roles in general metabolism and share physical similarities.

Amino acids are among the most suitable candidates for focused metabolomics as they are either ingested or synthesized endogenously and play essential physiological roles both as basic metabolites and metabolic regulators. To measure amino acids, plasma free amino acids (PFAAs), which abundantly circulate as a medium linking all organ systems, would be the most favorable target because their profiles have been known to be influenced by metabolic variations in specific organ systems induced by specific diseases [18], [19], [20], [21]. Additionally, plasma samples can be collected easily from patients.

**Figure 1:**
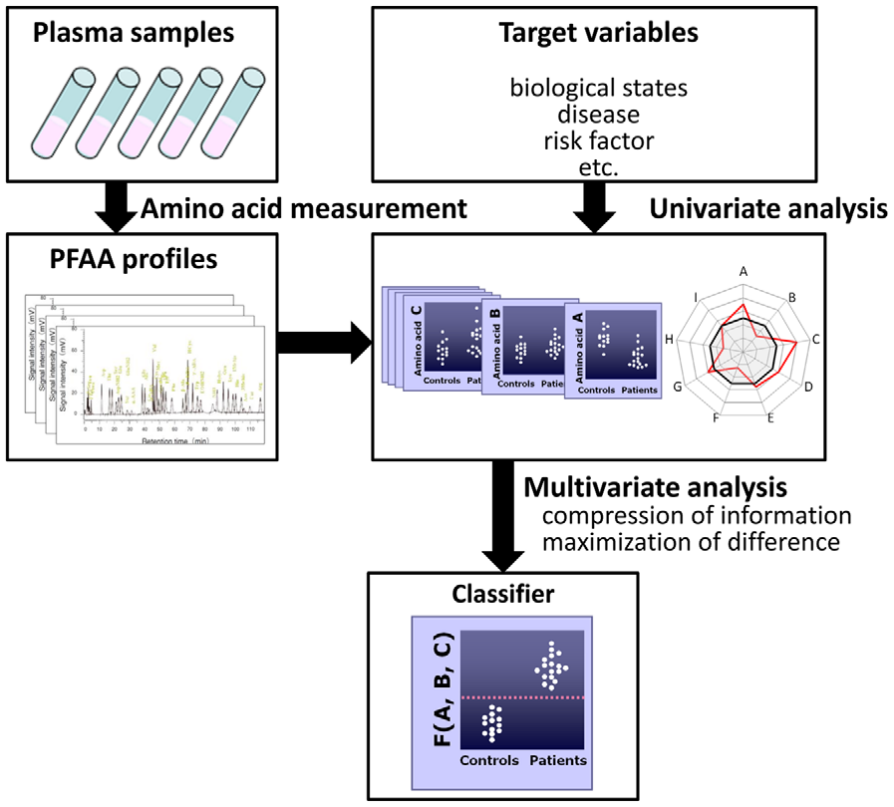
Concept of the generation of “AminoIndex technology”. At the top of the diagram, PFAA concentrations are measured for each subject. In the middle, target variables and univariate analysis of PFAA profiles are represented. At the bottom, an estimation of the classifier with optimized discriminating power using multivariate analysis is presented.

Several investigators have also reported changes in PFAA profiles in cancer patients [22], [23], [24], [25], [26], [27], [28]. However, despite evidence of a relationship between PFAA profiles and some types of cancer, few studies have explored the use of PFAA profiles for diagnosis because, although PFAA profiles differ significantly between patients, the differences in individual amino acids do not always provide sufficient discrimination abilities by themselves [24], [29], [30]. To address this issue, we previously constructed and tested a diagnostic index based on PFAA concentrations, known as the “AminoIndex technology” [29], [30], [31], [32], [33], to compress multidimensional information from PFAA profiles into single dimension and maximize the differences between patients and controls (Figure 1). We obtained preliminary data on the efficacy of the “AminoIndex technology” for the early detection of colorectal, breast, and lung cancers in approximately 150 samples from a single medical institute [29], [30].

Moreover, technologies have recently been developed to analyze amino acids with high accuracy. For example, we developed a method to measure PFAA profiles using high-performance liquid chromatography (HPLC)–electrospray ionization (ESI)–mass spectrometry (MS) [34], [35], [36].

The present study aimed to determine the possibility of PFAA profiling for cancer diagnosis using a large number of samples from multiple medical institutes. We measured the PFAA profiles of approximately 200 cancer patients from three different institutes each with one of the following five types of cancer: lung, gastric, colorectal (CRC), breast, or prostate cancer. Patients were compared to five times sizes of gender- and age-matched controls also used in this study. We then compared the alterations in the PFAA profiles between the cancer patients and the controls using univariate and multivariate analyses. As a result, significant alterations in PFAA profiles were observed in cancer patients in comparison to control subjects. We demonstrated two types of alterations in PFAA profiles in cancer patients: some differences reflected the metabolic changes common to many cancers, while others were specific to each type of cancer. We also found that both common and cancer type-specific alterations in PFAA profiles were observed even in the patients with early stage cancer. Furthermore, using a large number of samples allowed us to verify the robustness of PFAA profiling for the early detection of various cancers.

## Materials and Methods

### Ethics

The study was conducted in accordance with the Declaration of Helsinki, and the protocol was approved by the ethics committees of the Kanagawa Cancer Center, the Osaka Medical Center for Cancer and Cardiovascular Diseases, the Okayama University Hospital, the Yokohama City University Medical Center, the Gunma Prefectural Cancer Center, the Shizuoka Prefectural Cancer Center, the Chiba Prefectural Cancer Center, the Yokohama Municipal Citizen's Hospital, the Yokohama Minami Kyosai Hospital, the Kanagawa Health Service Association, the Kameda Medical Center Makuhari, and the Mitsui Memorial Hospital. All subjects gave their written informed consent for inclusion before they participated in the study. All data were analyzed anonymously throughout the study.

### Subjects

Data from Japanese patients with lung cancer (LC), gastric cancer (GC), colorectal cancer (CRC), breast cancer (BC), and prostate cancer (PC) were analyzed in this study. The patients had been histologically diagnosed with primary cancer at various Japanese medical institutes between 2006 and 2009. The LC patients were recruited from the Osaka Medical Center for Cancer and Cardiovascular Diseases, the Chiba Prefectural Cancer Center, the Kanagawa Cancer Center, and the Gunma Prefectural Cancer Center. The GC patients were recruited from the Okayama University Hospital, the Gunma Prefectural Cancer Center, and the Shizuoka Prefectural Cancer Center. The CRC patients were recruited from the Kanagawa Cancer Center, the Shizuoka Prefectural Cancer Center, and the Gunma Prefectural Cancer Center. The BC patients were recruited from the Yokohama City University Medical Center, the Kanagawa Cancer Center, and the Gunma Prefectural Cancer Center. The PC patients were recruited from the Kanagawa Cancer Center, the Yokohama Municipal Citizen's Hospital, the Yokohama Minami Kyosai Hospital, and the Gunma Prefectural Cancer Center. Control subjects with no apparent cancer were chosen from among those undergoing comprehensive medical examinations at three different Japanese medical institutes (the Center for Multiphasic Health Testing and Services of the Mitsui Memorial Hospital, the Kameda Medical Center Makuhari, and the Kanagawa Health Service Association) between 2008 and 2009.

Colonic polyp patients were recruited from among those undergoing endoscopic polypectomy at the Kameda Medical Center Makuhari between 2006 and 2008.

For the purposes of data analysis, the patients were assigned to five groups based on their primary cancer diagnoses (∼140–200 patients per group), and five age- and gender-matched control groups were also established (Table 1). Data sets for all of the cancer patients and controls, as well as all cancer patients stratified by gender, were also analyzed.

**Table 1:** Demographic and clinical characteristics of subjects.

Data set		LC		GC		CRC		BC		PC	
		Patients	Controls	Patients	Controls	Patients	Controls	Patients	Controls	Patients	Controls
Size	Total	200	996	199	985	199	995	196	976	134	666
	M/F	125/75	635/371	126/73	626/359	114/85	570/425	0/196	0/976	134/0	666/0
Age	Mean	65.0^a^	63.2	64.8^a^	62.9	63.7	62.4	55.3	54.5	69.4^c^	65.8
	(SD)	(10.0)	(9.2)	(10.8)	(9.7)	(9.5)	(9.5)	(12.6)	(11.1)	(6.7)	(6.1)
BMI	Mean	22.5	22.9	22.7	22.8	23.0	22.8	22.4	22.0	23.4	23.4
	(SD)	(3.8)	(3.0)	(3.2)	(3.0)	(3.7)	(3.0)	(3.4)	(3.5)	(2.7)	(2.5)
Stage	0	-		-		8		26	-	-	
	I(A)	29		120		63		75		0	
	II(B)	16		29		48		73		95	
	III(C)	54		26		59		13		19	
	IV(D)	28		24		19		0		15	
	Uncharacterized	1		0		2		9		5	

a *p*<0.05,

c *p*<0.001.

For LC, GC, CRC, and BC, cancer stages were determined according to the International Union Against Cancer TNM Classification of Malignant Tumors, 6th edition [38], and for PC, cancer stages were determined according to Jewett staging system [39].

### PFAA measurement

Blood samples were collected from the controls and the patients prior to any medical treatment. Blood samples (5 ml) were collected from forearm veins after overnight fasting in tubes containing ethylenediaminetetraacetic acid (EDTA; Termo, Tokyo, Japan) and were immediately placed on ice. Plasma was prepared by centrifugation at 3,000 rpm at 4°C for 15 min and then stored at −80°C until analysis. After the plasma collection, all samples were stored and processed at the Institute for Innovation of the Ajinomoto Co., Inc. (Kawasaki, Japan). To reduce any bias introduced prior to analysis, samples were analyzed in random order. The plasma samples were deproteinized using acetonitrile at a final concentration of 80% before measurement. The amino-acid concentrations in the plasma were measured by HPLC–ESI–MS, followed by precolumn derivatization. The analytical methods used were as described previously [34], [35], [36].

Among the 20 genetically-encoded amino acids, glutamate (Glu), aspartate (Asp), and cysteine (Cys) were excluded from the analysis because they are unstable in blood. Citrulline (Cit) and ornithine (Orn) were measured instead because they are relatively abundant in blood and are known to play important roles in metabolism. The following 19 amino acids and related molecules were therefore measured and analyzed: alanine (Ala), arginine (Arg), asparagine (Asn), Cit, glutamine (Gln), glycine (Gly), histidine (His), isoleucine (Ile), leucine (Leu), lysine (Lys), methionine (Met), Orn, phenylalanine (Phe), proline (Pro), serine (Ser), threonine (Thr), tryptophan (Trp), tyrosine (Tyr), and valine (Val).

Two metrics were made for each of the 19 amino acids including the absolute concentration of each amino acid, which directly reflected its availability and consumption, and the ratios associated with the specific metabolic status in each organ. The concentrations of the amino acids in the plasma were expressed in µM, and the ratios of the amino acid concentrations were expressed by the follow equation:
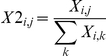
where *X2_i,j_* is ratio of the amino-acid concentration of the j-th amino acid of i-th subject, and *X_i,j_* is the plasma concentration (µM) of the j-th amino acid of i-th subject.

### Statistical analysis

Two types of metric were used for each data set for analysis using either the amino-acid concentration or the ratio as explanatory variables.

#### Mean and SD

The mean amino-acid concentrations ± standard deviations (SDs) were calculated to determine summarized PFAA profiles for both patients and controls.

#### Mann-Whitney U-test

The Mann-Whitney *U*-test was used to assess significant differences of the plasma amino-acid concentrations between the patients and the controls.

#### ROC analysis

Receiver-operator characteristic (ROC) curve analyses were performed to determine the abilities of uni- and multi-variate analyses to discriminate between patients and controls. The patient labels were fixed as positive class labels. Therefore, an area under the ROC curve (AUC of ROC) value of <0.5 indicated that the amino acid level was lower in the patients than the controls, whereas an AUC of ROC value of >0.5 indicated that it was higher. The 95% confidence interval (95% CI) of AUC of ROC for the discrimination of patients based on amino acid concentrations and ratios was also estimated as described by Hanley and McNeil [37].

#### Two-way analysis of variance (ANOVA)

The two-way ANOVA was used to evaluate the effects of gender, age, and smoking status as potential confounding factors. The presence of cancer and gender were assumed to be independent factors, age was treated as a continuous predictor rather than a categorical predictor, and the interaction term between the presence of cancer and smoking status was analyzed.

#### Two-class linear discrimination analysis (LDA)

Linear discrimination analysis (LDA) with stepwise variable selection was performed to distinguish patients with each type of cancer from the control subjects, in which both the maximum and the minimum p-values for a term to be added or removed were set at 0.001.

#### Multi-class LDA for discrimination

LDA with stepwise variable selection was also performed to distinguish patients with a specific cancer from the complete data set containing all cancer patients stratified by gender (four kinds of cancer patients in each data set). Because the size of each group was smaller than that of two-class LDA, the maximum p-value for a term to be added was set at 0.05 and the minimum p-value for a term to be removed was set at 0.10. The Mahalanobis distance was used as a metric of classification. The accuracy was defined as the ratio of the correctly discriminated patients to the total number of patients with each cancer instead of AUC of ROC because ROC analysis could be applied only for two-class discrimination.

#### Leave one out cross—validation (LOOCV)

LOOCV was performed to correct potential over-optimization for obtained LDA models. Briefly, one sample was omitted from the study data set, and the LDA model was calculated for the remaining samples to estimate coefficients for each amino acid. The function values for the left-out sample were calculated based on the model. This process was repeated until every sample in the study data set had been left out once.

#### Conditional logistic-regression (c-logistic) analysis

C-logistic analysis was also performed to verify the effects of age and gender, potential confounding factors, on the discriminatory abilities of obtained LDA models to differentiate patients with each type of cancer from the controls.

#### Subgroup analysis

To assess the effects of cancer stage, each data set was divided into a sub-data set according to disease stage and including corresponding controls, and analyzed using the ROC analysis in each data set.

### Software

MATLAB (The Mathworks, Natick, MA) was used for the calculations of mean and SD, the Mann-Whitney *U*-test, ROC analysis, two-way ANOVA, LDAs, and LOOCV. GraphPad Prism (GraphPad Software, La Jolla, CA) was also used for the ROC curve analysis. LogXact (Cytel, Cambridge, MA) was used for the c-logistic analysis.

## Results

### Characteristics of subjects

Table 1 summarizes the characteristics of the subjects in this study. The data sets comprised 200 LC patients and 996 controls, 199 GC patients and 985 controls, 199 CRC patients and 995 controls, 198 BC patients and 976 controls, and 134 PC patients and 666 controls (Table 1). The sample size for each cancer type was greater than those in previous reports [25] and provided sufficient statistical power to test the robustness of the PFAA profiles for cancer diagnosis.

There were no significant differences in body mass index (BMI) among the data sets (Table 1). Weight loss due to malnutrition was therefore not expected to influence the results. Although significant differences in average age were observed among the data sets (LC, *p*<0.05; GC, *p*<0.05; and PC, *p*<0.001), the effects appeared to be relatively minor because the absolute values of these differences were small (Table 1).

For LC, GC, CRC, and BC, disease stages were determined according to the Sixth Edition of the International Union Against Cancer (UICC) Tumor–Node–Metastasis (TNM) Classification of Malignant Tumors [38]. For PC, the stage was determined according to the Jewett staging system [39]. For all types of cancer, a large proportion of the patients had early-stage disease. The fractions of patients at each stage according to type of cancer were as follows: ∼50% stage I, ∼10% stage II, ∼25% stage III, and ∼15% stage IV for LC; ∼60% stage I, ∼15% stage II, ∼13% stage III, and ∼12% stage IV for GC; ∼35% stages 0 and I, ∼25% stage II, ∼30% stage IV, and ∼10% stage IV for CRC; ∼5% stage 0, ∼25% stage I, ∼25% stage II, and ∼7% stage III for BC; and ∼75% stage B, ∼13% stage C, and ∼12% stage D for PC (Table 1).

The patients with each type of cancer could be further subdivided based on histological type (for LC, GC, CRC, and BC) or Gleason score (for PC), as is summarized in Table S1. The characteristics of 34 colonic polyp patients as well as the smoking status of patients are also summarized in Table S1.

### Shared PFAA profiles among cancers

**Figure 2:**
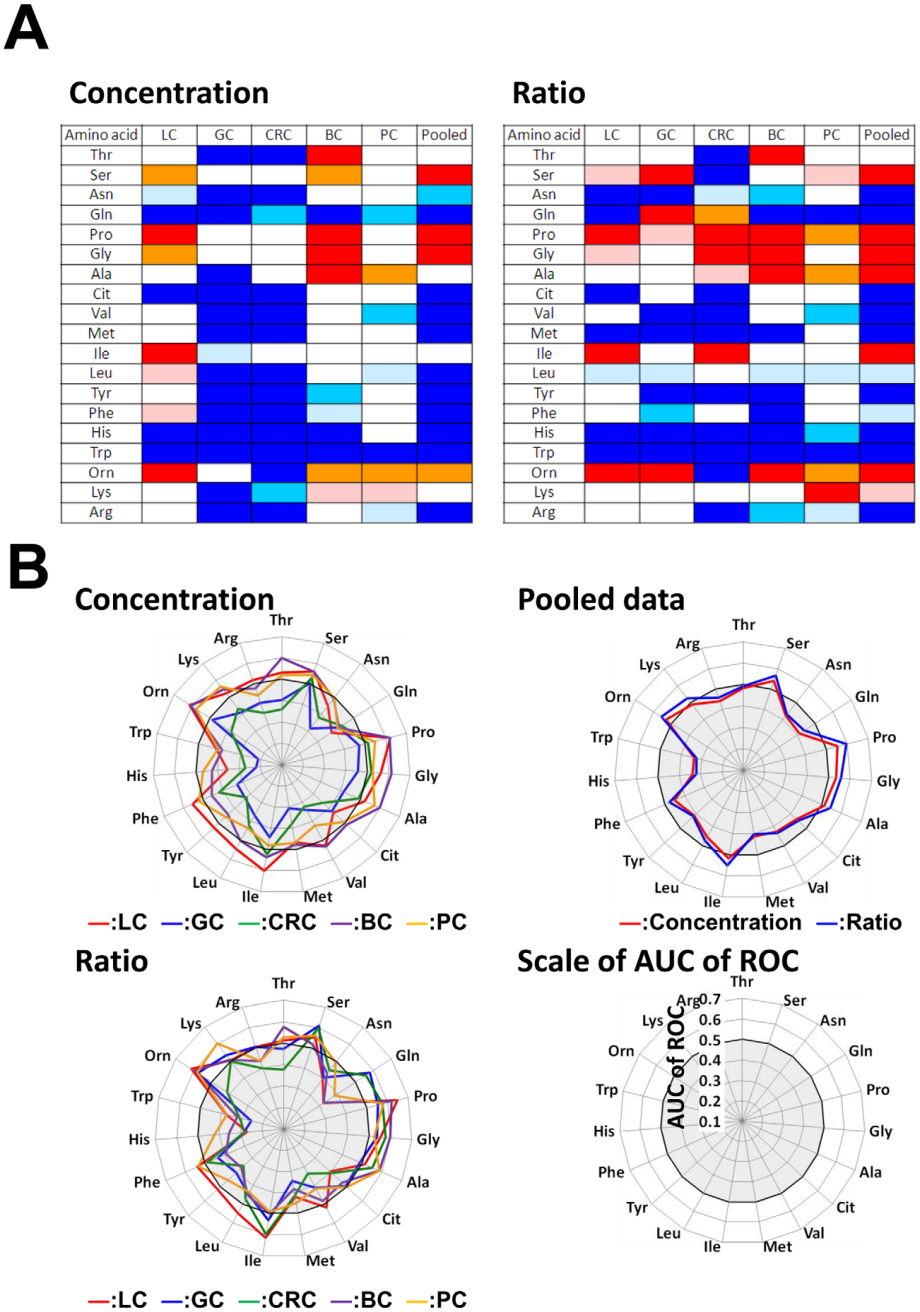
PFAA profiles of cancer patients. The results of the Mann-Whitney *U*-test (A) and receiver-operator characteristic (ROC) curve analysis (B) are indicated. A. Colored cells indicate that the concentration or ratio is increased in cancer patients at p<0.001 (red), p<0.01 (orange), and p<0.05 (pink), and decreased in cancer patients at p<0.001 (blue), p<0.01(sky blue), and p<0.05 (light blue), respectively. B. Axes show the AUC of ROC for each amino acid to discriminate patients from controls. Concentrations and ratios of each cancer patient and the pooled data set are indicated, respectively. Black bold lines indicate the point where the AUC of ROC = 0.5.

Univariate analysis was used to compare the PFAA profiles of the cancer patients and controls. The differences in the significance levels of each amino acid between the patients and the controls are shown in Figure 2A. The results of the ROC analysis are depicted in Figure 2B because the levels of significance depend on sample size. The concentrations and ratios of each amino acid profile for both patients and controls are shown in Tables S2. And the AUCs of ROC and their CIs of each amino acid are shown in Table S3 (concentration) and Table S4 (ratio), respectively.

Two-way ANOVA was used to evaluate the potential confounding effects of gender, age, and smoking status. Correcting for these factors did not greatly affect the significance levels of each amino acid, suggesting that their effects on the PFAA profiles were minor (Table S5).

The plasma concentrations of Gln, Trp, and His were significantly decreased in all of the cancers except PC, and none of the amino acids showed increased concentrations across all types of cancer (*p*<0.05). The ratios of Trp and His were significantly decreased, while those of Pro and Orn were increased, in all cancers (*p*<0.05) (Figure 2).

To further examine the shared traits among cancer patients, the PFAA profiles were compared using a pooled data set including all cancer patients and controls. Notably, the amino acids that were affected by this type of analysis had significant differences in both concentration and ratio: 11 amino acids (Asn, Gln, Cit, Val, Met, Leu, Tyr, Phe, His, Trp, and Arg) showed decreases, while four amino acids (Ser, Pro, Gly, and Orn) exhibited increases (Figure 2). Changes in Gln, Trp, His, Pro, and Orn were detected in the analysis for all types of cancer. Alterations in these amino acids might therefore reflect characteristic changes in metabolism that are common to all cancers.

### Specific PFAA profiles for each cancer

In addition to the changes that were common to all of the cancers, we detected alterations in PFAA profiles that were specific to each disease type (Figure 2). Overall, the concentrations of most amino acids were decreased in GC and CRC patients, whereas no clear trends in amino acid concentrations were observed in the other groups (Figure 2). Furthermore, some of the amino acids showed opposite trends in different types of cancer. For example, the concentrations of Thr were decreased in GC and CRC patients, but increased in BC patients (Figure 2). These variations in the PFAA profiles might reflect specific characteristics of each cancer, in contrast to the limited set of amino acids that are responsible for the metabolic changes shared by all cancers.

### Changes in PFAA profiles in early-stage cancers

**Figure 3:**
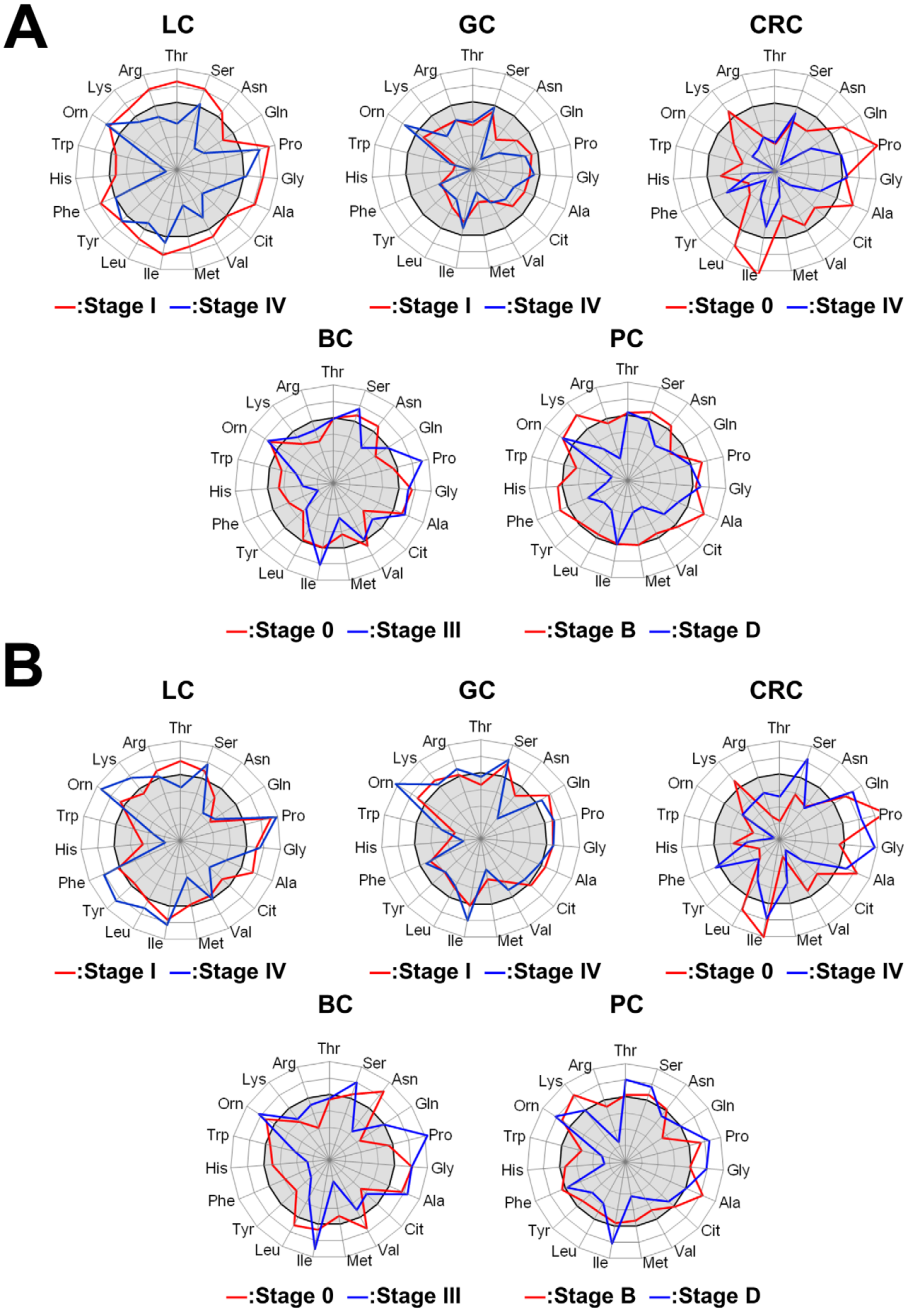
PFAA profiles of early- and advanced-stage cancer patients. The axes show the AUC of ROC for each amino acid for discriminating patients from controls. A. Comparison of concentrations of cancer patients and controls. B. Comparison of ratios of cancer patients and controls. Scale as described for Figure 2. For LC, GC, CRC, and BC, cancer stages were determined according to the International Union Against Cancer TNM Classification of Malignant Tumors, 6th edition [38], and for PC, cancer stages were determined according to Jewett staging system [39].

Although alterations in the PFAA profiles of cachexic patients with advanced cancer have been well documented, few reports have considered early-stage patients. However, a large fraction of the cancer patients in the current data set were in the early stages of disease (Table 1). The differences in PFAA profiles according to disease stage were therefore examined for each cancer (Figure 3, Figure S1, Table S3, Table S4).

Notably, alterations in the PFAA profiles were detected in all patients, including those in the early stages of disease, in the current study. All amino-acid concentrations and ratios were drastically decreased in early stage disease patients, regardless of the subsequent progression. In particular, significant decreases of each amino acid concentration were observed in GC and CRC patients (Figure 3A), and changes in each ratio were notable in all of the cancer patients (Figure 3B).

Early-stage cancer patients are generally asymptomatic. Moreover, most of the subjects in the present study did not show significant weight loss (a symptom typical of cachectic patients) (Table 1), anorexia, or decreases in serum albumin concentrations (data not shown). The changes in the PFAA profiles in cancer patients therefore appeared to be independent of any effects caused by poor nutrition resulting from tumor progression.

### Discriminating cancer patients and controls by PFAA profiles

The results of the univariate analyses suggested that cancer patients and controls could be discriminated using multivariate analysis. By assuming that the presence of cancer and the concentrations or ratios of the PFAA profiles were objective and explanatory variables, respectively, LDA was able to distinguish cancer patients from the corresponding controls with variable selection. The results of variable selection are indicated in Table 2 (concentration) and Table S6 (ratio), respectively.

**Table 2:** Variables incorporated into LDA and c-logistic models using concentrations as explanatory variables.

Amino acid	LC		GC		CRC		BC		PC		Pooled	
	LDA	C-logit	LDA	C-logit	LDA	C-logit	LDA	C-logit	LDA	C-logit	LDA	C-logit
Thr							+++	+++			+++	+++
Ser	+++	+++			+++	+++					+++	+++
Asn												
Gln	−−−	−−−					−−−	−−−	−−−	−−−	−−−	−−−
Pro	+++	+++									+++	+++
Gly							+++	++				
Ala					+++	+++	+++	+++	+++	+++	+++	+++
Cit	−−−	−−−	−−−	−							−−−	
Val	−−−	−	−−−	−−	−−−	−−−			−−−	−−−	−−−	−−−
Met											−−−	−−−
Ile	+++	+++	+++	+	+++	+++			+++	++	+++	+++
Leu					+++	+++					+++	++
Tyr					−−−	−−−	−−−	−−				
Phe	+++	+++									+++	+++
His	−−−	−−−	−−−	−−−	−−−	−−−					−−−	−−−
Trp	−−−	−−−	−−−	−−−	−−−	−−	−−−	−−−	−−−	−−−	−−−	−−−
Orn	+++	+++					+++	+++	+++	+++	+++	+++
Lys			+++	+++	+++	+++			+++	+++	+++	+++
Arg					−−−	−−−			−−−	−−−	−−−	−−−

+, ++, +++: positive coefficients in the model.

−, −−, −−−: negative coefficients in the model.

+,−: p<0.05, ++,−−: p<0.01, +++,−−−: p<0.001.

The discrimination abilities for each cancer patient were evaluated using the AUC of ROC of the discriminate score and were found to be >0.75 in all cases (Table 3 and Table S7). In concrete analysis, AUCs for the discrimination of patients based on the amino acid concentrations and ratios, respectively, were also estimated as follows: 0.802 (95% CI: 0.766∼0.838) and 0.802 (95% CI: 0.767∼0.837) for LC; 0.849 (95% CI: 0.816∼0.882) and 0.816 (95% CI: 0.780∼0.852) for GC; 0.874 (95% CI: 0.842∼0.906) and 0.881 (95% CI: 0.851∼0.910) for CRC; 0.778 (95% CI: 0.741∼0.815) and 0.778 (95% CI: 0.741∼0.815) for BC; and 0.783 (95% CI: 0.740∼0.826) and 0.779 (95% CI: 0.740∼0.819) for PC (Table 3 and Table S7). The discriminate analysis was therefore able to adequately distinguish between different types of patient cancer.

**Table 3:** Discrimination performance of LDA and c-logistic models using concentrations as explanatory variables.

Model	Subjects		LC	GC	CRC	BC	PC	Pooled
LDA	All	AUC	0.802	0.849	0.874	0.778	0.783	0.796
		CI	(0.766∼0.836)	(0.816∼0.882)	(0.842∼0.906)	(0.741∼0.815)	(0.740∼0.826)	(0.779∼0.814)
	LOOCV	AUC	0.792	0.845	0.868	0.769	0.767	0.793
	Stage 0 patients	AUC	-	-	0.903	0.813		
		CI			(0.807∼1.00)	(0.726∼0.900)		
	Stage I patients	AUC	0.752	0.859	0.859	0.754		
		CI	(0.698∼0.805)	(0.820∼0.898)	(0.800∼0.918)	(0.692∼0.817)		
	Stage II(B) patients	AUC	0.870	0.829	0.921	0.786	0.764	
		CI	(0.772∼0.969)	(0.726∼0.933)	(0.877∼0.954)	(0.727∼0.847)	(0.710∼0.819)	
	Stage III(C) patients	AUC	0.844	0.834	0.817	0.755	0.777	
		CI	(0.780∼0.908)	(0.748∼0.920)	(0.743∼0.892)	(0.621∼0.889)	(0.669∼0.885)	
	Stage IV(D) patients	AUC	0.901	0.843	0.950	-	0.873	
		CI	(0.837∼0.966)	(0.734∼0.951)	(0.895∼1.00)		(0.771∼0.974)	
C-logit	All	AUC	0.806	0.850	0.876	0.776	0.786	0.798
		CI	(0.771∼0.841)	(0.816∼0.883)	(0.845∼0.907)	(0.739∼0.812)	(0.743∼0.829)	(0.780∼0.815)

Variable selection was also performed for each cancer patient. Eight amino acids were selected in more than two of the five kinds of cancers: Gln, Ala, Val, Ile, His, Trp, Orn, and Lys for the concentrations (Table 2A); and Ser, Gln, Val, Met, His, Trp, Lys, and Arg for the ratios (Table S6). Four of the amino acids (Gln, Val, His, and Trp) among each set were selected for both explanatory variables (Table 2 and Table S6). These amino acids were similar to those associated with all types of cancer as indicated by the univariate analysis (Gln, Trp, His, Pro, and Orn).

On the other hand, some amino acids incorporated into the LDA model were not identified as significant amino acids by the univariate analysis. For example, the Val concentration did not show a significant alteration in the univariate analysis (Figure 2A), but it was incorporated into the LDA model (Table 2). Because plasma concentrations of each amino acid are metabolically connected to each other, there might be a potential correlation that cannot be detected by the univariate analysis alone. Indeed, Spearman's partial correlation coefficient between Val and cancer (or not) was −0.127 (p<0.001), while the correlation coefficient between these two factors was 0.035 (not significant). Therefore, this suggested that the obtained LDA model reflected the metabolic network of PFAAs, which were not apparent thorough univariate analysis.

Because the obtained results may have been over-optimized, LOOCV was carried out to generate an unbiased analysis. This produced AUCs similar to those obtained for LDA, suggesting that there was no obvious over-optimization in the obtained LDA models (Table 3 and Table S7).

Subgroup analyses of divided data sets according to cancer stage, including corresponding controls, were then performed to assess the ability of PFAA profiles to distinguish between stages of cancer for each type of disease. In any stage of each cancer, the AUC of ROC was found to be higher than 0.75, suggesting that the obtained LDA models would thus be expected to be effective in detecting early as well as advanced stage cancers (Table 3 and Table S7).

The discrimination abilities for all cancer patients were also evaluated. The AUCs of ROC for both concentrations and ratios were 0.796 (95% CI: 0.779∼0.814) and 0.785 (95% CI: 0.767∼0.803), respectively (Table 3 and Table S7). Notably, most of the 19 amino acids were statistically selected for these discriminations: 16 for the concentrations and 12 for the ratios. Even using a rough classification, regardless of the type of cancer, it was possible to discriminate between patients and controls with high accuracy, and the overall contributions of numerous amino acids might reflect the large-scale characteristic changes associated with cancer metabolism.

A c-logistic analysis using matching factors (gender and age) was performed for each data set to evaluate and correct for potential confounding factors. Note that we used the combinations of amino acids obtained from the LDA models as explanatory variables. Although the c-logistic analysis was performed using all of the significant variables identified by the univariate analysis, the amino acids identified in the LDA were utilized to correct for potential confounding factors more adequately (data not shown). Both the levels of significance (Table 2 and Table S6) and the discrimination abilities (Table 3 and Table S7) were not significantly altered by correcting for the potentially confounding factors, suggesting that these results were independent of gender and age effects.

To evaluate patients with non-neoplastic diseases, the PFAA profiles of colonic polyp patients were substituted into the LDA model for CRC. Most of the colonic polyp patients (31/34, 91.2%) were classified into the control group for the concentrations and ratios of both models, suggesting that the obtained models could discriminate CRC patients specifically.

### Discrimination between cancer types by PFAA profiles

In addition to differentiating between patients with each type of cancer and the controls, discrimination among patients within each cancer group was also performed by separating all the cancer patients into each disease subtype according to gender. This was done because the results of the present analyses identified changes in PFAA profiles that were common to all types of cancer as well as those specific to individual cancers.

The accuracies of all discriminant analyses using amino acid concentrations as explanatory variables were close to or better than 50% both in male patients (Table 4) and female patients (Table 5) data set. The discrimination accuracy among cancer patients was less than that between patients and controls. Six amino acids (Gly, Cit, Val, Tyr, Trp, and Arg) were commonly selected in these analyses, regardless of gender (data not shown). An additional six amino acids (Gln, Met, Leu, His, Orn, and Lys) were selected in the male patient data set, and four (Thr, Ser, Ile, and Phe) were selected in the female patient data set (data not shown). Five of the 16 amino acids listed above were selected in the discrimination between patients and controls, while the remainder might have been responsible for the characteristic features of each cancer.

**Table 4:** Multiclass discriminant analyses of male cancer patients using concentrations as explanatory variables.

		Patients with:			
		LC	GC	CRC	PC
Discriminated as:	LC	**72(69)**	*19(22)*	*12(13)*	*26(26)*
	GC	*18(19)*	**58(52)**	*16(17)*	*25(25)*
	CRC	*13(14)*	*25(28)*	**71(69)**	*16(17)*
	PC	*22(23)*	*24(24)*	*15(15)*	**67(66)**
	Total	125	126	114	134
	Accuracy	**57.6%(55.2%)**	**46.0%(41.3%)**	**62.3%(60.5%)**	**50.0%(49.3%)**

The numbers in the blanket indicate the results of LOOCV.

**Table 5:** Multiclass discriminant analyses of female cancer patients using concentrations as explanatory variables.

		Patients with:			
		LC	GC	CRC	BC
Discriminated as:	LC	**41(37)**	*4(6)*	*8(11)*	*43(44)*
	GC	*13(14)*	**40(38)**	*15(16)*	*30(30)*
	CRC	*6(8)*	*13(13)*	**52(47)**	*17(17)*
	BC	*15(16)*	*16(16)*	*10(11)*	**106(105)**
	Total	75	73	85	196
	Accuracy	**54.7%(49.3%)**	**54.8%(52.1%)**	**61.2%(55.2%)**	**54.1(53.6%)**

The numbers in the blanket indicate the results of LOOCV.

The accuracies were similar between the analyses using ratios as explanatory variables and those using concentrations both in male patients (Table S8) and female patients (Table S9). Seven amino acids (Gln, Cit, Val, Tyr, Trp, Lys, and Arg) were commonly selected regardless of gender in these analyses (data not shown). An additional four amino acids (Ala, Met, Leu, and His) were selected in the male patient data set, and four (Thr, Ser, Ile, Orn) were selected in the female patient data set (data not shown). Five amino acids (Cit, Val, Tyr, Trp, and Arg) from each set were selected for both explanatory variables, suggesting that the changes to the respective PFAAs were specific to certain types of cancer.

LOOCV was also carried out and resulted in similar accuracies for the discrimination analyses, suggesting that there was no obvious over-optimization in the obtained models (Table 4, Table 5, Table S8 and Table S9).

## Discussion

The present study demonstrated the use of PFAA profiling as a focused metabolomics approach for the early detection of patients with any of five types of cancer. Combining novel analytical techniques and both univariate and multivariate statistical analyses, previously unknown aspects of amino acid metabolism in humans have been revealed. The sample size in the present study was considerably larger than those reported previously [25], [29], [30], and provided sufficient statistical power to test the robustness of PFAA profiling for cancer diagnosis. We also demonstrated the possibility of detecting cancers, both specifically and broadly, using multivariate analysis to compress the PFAA profile data, even for patients with early stage cancer.

In the previous studies, the alterations in PFAA profiles in cancer patients sometimes seem inconsistent[22], [23], [24], [25], [26], [27], [28], [29], [30], and some discrepancies existed between our current study and those reported in the literature [25]. This discrepancy may be due not only to sample size and the varying predominance of early stage cancers but also to some other factors such as amino acid measurement methods. On the other hand, alterations in the PFAA profiles in our present study were consistent with the results of our previous studies, in which samples were collected from a single medical institute [29], [30]. Furthermore, there are also many similarities between our results and those of previous studies. For example, decreases in His and Gln levels, which have been observed broadly in previous reports, and increases in Pro and Ala levels in BC are consistent with our findings [25].

Cancer is expected to become the leading cause of death worldwide within a few years. Therefore, it is crucial that methods for the prevention, early detection, and treatment of cancers should be implemented to reduce mortality. Various screening methods have been established for the cancers included in our study. However, the high specificity of these methods means that subjects must undergo each screening examination separately, which can be expensive and time consuming. These examinations can also impose a physical and/or mental burden upon subjects, which can lead to avoidance. By contrast, the method described in the present study involves a relatively simple plasma assay and imposes a low physical burden on subjects. This method could also be used as versatile health assessment as other diseases in which PFAA profiles can be altered, such as diabetes[18], hepatic failure[19], and renal failure[21], can also be evaluated.

It should be noted that the models derived from this case-control study could not be used directly to make further observations or predictions, despite providing a preliminary demonstration of the potentially high value of this method for cancer discrimination. Further investigations, including model construction and validation using cohorts with larger sample sizes, are in progress to clarify the clinical utility of this approach. Moreover, the possibility of continuous PFAA profiling as a means to determine prognosis after surgery or chemotherapy is also being investigated.

Our investigation demonstrated two types of alterations in PFAA profiles of cancer patients: those in a limited set of amino acids reflecting metabolic changes common to many cancers; and those in a larger group of amino acids representing metabolic characteristics specific to each cancer. Alterations in PFAA profiles were observed even in patients with early-stage cancer, most of whom had no apparent symptoms. This strongly suggested that the alterations in PFAA profiles identified in the current study were independent of the effects of poor nutrition caused by tumor progression.

Many previous reports have shown that metabolism, including that of amino acids, is notably altered in cancer cells [3], [13], [40] and that changes in PFAA profiles can also occur [22], [24], [25], [26], [27], [28], [29], [30], especially in cachexic patients with advanced cancer [23], [25]. Among whole metabolites, amino acids have been frequently identified as having associations with cancer in other studies [10], [13], [41], [42], [43]. The current study demonstrated that mechanisms other than malnutrition can drive the changes in PFAA profiles.

Besides cancer-dependent malnutrition, significant decreases in PFAA concentrations and various indicators of nutritional status such as BMI and serum albumin levels are observed in cancer-independent cachexia [44], [45], [46]. In the present study, no apparent decreases in those indicators were observed, strongly suggesting that alterations in PFAA were also independent of nutritional status mediated by factors not related to cancer.

Nevertheless, it remains unclear how the metabolic changes occurring in cancer patients affect the PFAA profile of the whole body, even in patients with early-stage tumors. To clarify the relationship between carcinogenesis and changes in PFAA profiles, we are further investigating the contribution of local effects caused by cancer cell metabolism and the systemic responses of the immune system against tumors or factors released by cancer cells.

Changes in metabolism can be detected in cancer cells even in early-stage patients. Hirayama *et al.* reported no significant correlation between the levels of cancer cell metabolites, including several amino acids, and the tumor stage [13]. The metabolism of Trp is of particular interest because it was identified as one of the most important amino acids in relation to cancer progression in our study. Overexpression of indoleamine-2,3- dioxygenase (IDO), the first enzyme in the kynurenine Trp metabolism pathway in humans, has been reported in cancer cells [47]. IDO is induced in many different tumors and has been suggested to play a role in cancer-mediated evasion of the immune system [47], [48], [49], [50].

Arg, Orn, Cit, and Pro are known to be closely related to immune function. For example, Qiu *et al.* reported an association between the urea cycle and metabolic alterations in CRC patients and found no correlation between the metabolite profile and cancer progression [43]. Cancer cells also release factors that can alter general physical conditions. For example, the transcriptional regulatory molecule high-mobility group B1 (HMGB1) was recently shown to regulate cancer-cell tumorigenesis, expansion, and invasion [51], [52], [53].

Further elucidation of these mechanisms might allow for the development of both static and dynamic models of carcinogenesis through system analysis [31]. Recently, computer-aided studies have been reported that integrate hierarchical ‘omics’ datasets for the systemic understanding of metabolic phenotypes to reconstruct the regulatory network from physiological data by means of system analysis. System analysis of cancer patients based on whole body amino acid metabolism could reveal information concerning the nature of a disease and help to establish strategies for its prevention, early detection, prognosis, monitoring, and treatment.

In contrast to many similar efforts to detect biomarkers of disease as single specific molecules (DNA, microRNA, proteins, peptides, or metabolites) in peripheral blood, our approach was to focus on the metabolic status, which is indicative of multivariate function, using non-specific metabolites. Therefore, we believe that our method is superior to those used in other studies, both in versatility and efficiency, because only one amino acid measurement can be applied for detection of various disease states (i.e., renal failure, hepatic failure, and nutritional status).

## Supporting Information

Figure S1 **PFAA profiles of cancer patients stratified by progression stage.** The axes show the AUC of ROC for each amino acid for discriminating patients from controls. A. Comparison of concentrations of cancer patients and controls. B. Comparison of ratios of cancer patients and controls. Scale as described for Figure 2. For LC, GC, CRC, and BC, cancer stages were determined according to the International Union Against Cancer TNM Classification of Malignant Tumors, 6th edition [38], and for PC, cancer stages were determined according to Jewett staging system [39].
(TIF)

Table S1 **Detailed demographic and clinical characteristics of subjects.** a: *p*<0.05, c: *p*<0.001 *: For LC, GC, CRC, and BC, cancer stages were determined according to the International Union Against Cancer TNM Classification of Malignant Tumors, 6th edition [38], and for PC, cancer stages were determined according to Jewett staging system [39].
(XLS)

Table S2 **PFAA profiles of cancer patients and controls.**
(XLS)

Table S3 **AUCs of ROC of each amino acid concentration for discrimination for cancer patients from controls.**
(XLS)

Table S4 **AUCs of ROC of each amino acid ratio for discrimination for cancer patients from controls.** AUCs were calculated using all patients and controls, and patiens and matched controls stratified by cancer stage.
(XLS)

Table S5 **Significance values for PFAA profiles for each data set by two-way ANOVA for the effects of cancer existence and other parameters.** Column headings indicate Mann-Whitney U-test of cancer existence (None), two-way ANOVA for the effects of cancer existence and gender (Gender), cancer existence and age (Age), and cancer existence and smoking status (Smoking).
(XLS)

Table S6 **Variables incorporated into LDA and c-logistic models using ratios as explanatory variables.** +, ++, +++: positive coefficients in the model −, −−, −−−: negative coefficients in the model +,−: p<0.05, ++,−−: p<0.01, +++,−−−: p<0.001.
(XLS)

Table S7 **Discrimination performance of LDA and c-logistic models using ratios as explanatory variables.**
(XLS)

Table S8 **Multiclass discriminant analyses of male cancer patients using ratios as explanatory variables.** The numbers in the blanket indicate the results of LOOCV.
(XLS)

Table S9 **Multiclass discriminant analyses of female cancer patients using ratios as explanatory variables.** The numbers in the blanket indicate the results of LOOCV.
(XLS)
