# Supplementary figures and images for: Plasma Free Amino Acid Profiling of Five Types of Cancer Patients and Its Application for Early Detection
Source: PLoS One. 2011 Sep 7;6(9):e24143. doi: 10.1371/journal.pone.0024143 (PMC3168486; doi:10.1371/journal.pone.0024143)

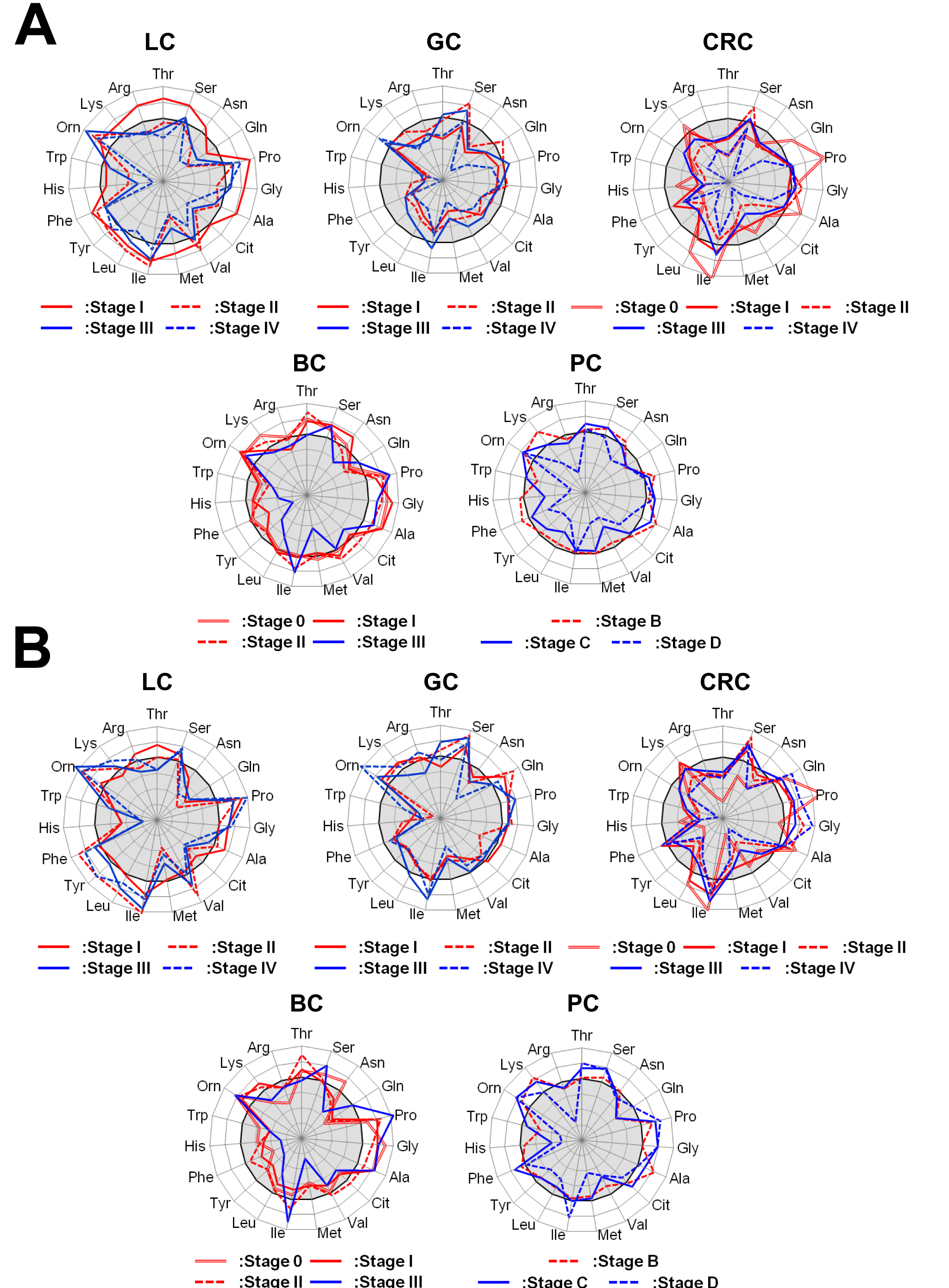

Supplement: Figure S1 — PFAA profiles of cancer patients stratified by progression stage. The axes show the AUC of ROC for each amino acid for discriminating patients from controls. A. Comparison of concentrations of cancer patients and controls. B. Comparison of ratios of cancer patients and controls. Scale as described for Figure 2. For LC, GC, CRC, and BC, cancer stages were determined according to the International Union Against Cancer TNM Classification of Malignant Tumors, 6th edition [38], and for PC, cancer stages were determined according to Jewett staging system [39]. (TIF) [file pone.0024143.s001.tif]
